# 1509. Clinical Features and Outcomes of COVID-19 in People Living with HIV in South Korea: a Nationwide Population-based Cohort Study

**DOI:** 10.1093/ofid/ofad500.1344

**Published:** 2023-11-27

**Authors:** Young Kyung Yoon, Jeong Yeon Kim, Yujin Jeong, Hyong Gin Ahn, Jin Woong Suh, Sun Bean Kim, Jang Wook Sohn

**Affiliations:** Korea University College of Medicine, Seoul, Seoul-t'ukpyolsi, Republic of Korea; Korea University College of Medicine, Seoul, Seoul-t'ukpyolsi, Republic of Korea; Korea University College of Medicine, Seoul, Seoul-t'ukpyolsi, Republic of Korea; Korea University College of Medicine, Seoul, Seoul-t'ukpyolsi, Republic of Korea; Korea University College of Medicine, Seoul, Seoul-t'ukpyolsi, Republic of Korea; Korea University, Seoul, Seoul-t'ukpyolsi, Republic of Korea; Korea University College of Medicine, Seoul, Seoul-t'ukpyolsi, Republic of Korea

## Abstract

**Background:**

The aim of this study was to assess whether people living with HIV were at increased risk of severe or critical COVID-19, compared with HIV-negative individuals with COVID-19.

**Methods:**

This nationwide descriptive epidemiological study was performed in South Korea between January 2020 and February 2022. Demographic and clinical characteristics were analyzed using National Health Insurance claims data collected through the Health Insurance Review and Assessment service (HIRA). We performed descriptive statistics and Cox proportional hazards regression analysis using the inverse probability weighting (IPW) to compare clinical features and outcomes between the HIV-positive and the HIV-negative COVID-19 patients.

**Results:**

The database contains claim records for 3,653,808 individuals who were diagnosed with COVID-19 and for whom hospitals issued claims to the HIRA. Of them, 1,311 subjects (0.04%) were people living with HIV. The proportion of male patients in the HIV-positive group was significantly higher than that in the HIV-negative group (88.6% vs. 46.3%, *P*< 0.001). The most common age groups were less than 20 years old in the HIV-positive group and 30 years old in the HIV-negative group, respectively. There was no significant difference in the mean Charlson's comorbidity index between the two groups. The mortality and morbidity rates in all COVID-19 patients were 0.24% and 15.7%, respectively. The hazard ratios (HRs) of hospitalization and in-hospital mortality in the HIV-positive group were 1.07 (95% CI 0.99-1.17; *P*=0.10) and 2.16 (95% CI 0.82-5.70; *P*=0.12) respectively, compared to those in the HIV-negative group. However, the odds ratios (ORs) of oxygen therapy (OR 2.82; 95% CI 2.26-3.52; *P*< 0.001), use of vasopressors or inotropes (OR 4.74; 95% CI 3.07-7.32; *P*< 0.001), and renal replacement therapy (OR 15.35; 95% CI 5.94-39.68; *P*< 0.001) were higher in the HIV-positive group, compared to those in the HIV-negative group.

**Conclusion:**

In conclusion, people living with HIV were not found to be at higher risk for mortality of COVID-19. However, they had a significantly higher risk of severe or critical COVID-19 than HIV-negative individuals. Our findings suggest the need for an immunization policy that prioritizes vaccination with the COVID-19 vaccine for people living with HIV.

**Disclosures:**

**All Authors**: No reported disclosures

